# Phenolic Characterization, Antioxidant Activity, and Enzyme Inhibitory Properties of *Berberis thunbergii* DC. Leaves: A Valuable Source of Phenolic Acids

**DOI:** 10.3390/molecules24224171

**Published:** 2019-11-17

**Authors:** María del Pilar Fernández-Poyatos, Antonio Ruiz-Medina, Gokhan Zengin, Eulogio J. Llorent-Martínez

**Affiliations:** 1 Department of Physical and Analytical Chemistry, Faculty of Experimental Sciences, University of Jaén, Campus Las Lagunillas, E-23071 Jaén, Spain; mpoyatos@ujaen.es (M.d.P.F.-P.); anruiz@ujaen.es (A.R.-M.); 2 Department of Biology, Science Faculty, Selcuk University, Campus, Konya 42130, Turkey; gokhanzengin@selcuk.edu.tr

**Keywords:** *Berberis thunbergii*, chlorogenic acid, caffeoylquinic acid, antioxidant, enzyme inhibition, phytochemical, phenolic

## Abstract

*Berberis* species are known for their use in traditional medicine. Here, we report the phenolic composition and bioactivity of methanolic and aqueous extracts of *Berberis thunbergii* DC. leaves. The phenolic profiling and the quantitation of the main compounds were performed by high-performance liquid chromatography with diode array and mass spectrometry detections. The most abundant compounds in both extracts were caffeoylquinic acids (chlorogenic acid, particularly, with a concentration of 90.1–101.3 mg g^−1^ dried extract), followed by caffeoylglucaric acids and quercetin glycosides. Antioxidant and radical scavenging assays (phosphomolybdenum, DPPH, ABTS, CUPRAC, FRAP, metal chelating activity), as well as enzyme inhibitory assays (acetylcholinesterase, butyrylcholinesterase, tyrosinase, amylase, glucosidase, and lipase), were carried out to evaluate the potential bioactivity of *B. thunbergii*. The methanolic extract presented the highest antioxidant and radical scavenging values, in agreement with its higher phenolic content. Regarding enzyme inhibitory potential, the methanolic extract was also more potent than the aqueous one. Hence, *B. thunbergii* leaves represent a suitable candidate for the preparation of pharmaceutical or nutraceutical products.

## 1. Introduction

*Berberis* is a large genus of the Berberidaceae family, which is represented by around 500 species distributed worldwide. This genus consists of spiny evergreen shrubs with yellow wood and flowers, yellow or orange, appear alone or in racemes (3–6 mm long). Leaves on long shoots develop into three-spine thorns and short shoots with several leaves (1–10 cm long). Fruits are small berries, red or blue after ripening [1].

The medicinal properties of *Berberis* have been known and appreciated for thousands of years. Many of them are due to the presence of alkaloids with different pharmacological activities [2], being berberine one of the most active compounds [3]. Some species of *Berberis* contain predominant phenolic compounds in their leaves, such as chlorogenic acid and rutin [4].

The plants belonging to this genus are known for their antidiabetic properties [5]. Some species have shown antibacterial and antifungal activities [6] and have been used in traditional medicine to cure heart diseases, digestive ailments, and problems with the urinary tract [4,7]. In natural medicine, *Berberis* leaves are used mainly for colds and common ailments of the body [4]. Commercial teas from *B*. *buxifolia* and decoctions of *B*. *vulgaris* have been traditionally used; in fact, infusions obtained from berberine-producing plants are used for their antimicrobial, anti-inflammatory, and antiseptic properties [1,8]. In addition to its curative applications, various species of this genus are commonly used in some cuisines [9].

*Berberis thunbergii* DC., also known as Japanese barberry, is a dense, woody shrub with deciduous nature that can reach up to 2 m high and is often used as an ornamental plant due to the bright red tones of the leaves [10]. Native to Asia, it is also present in the USA and in several European countries [3,11]. *B. thunbergii* is also recognized as a healing plant in Asia. It has been reported to present positive biological effects on health, such as antioxidant, anti-inflammatory, antibacterial, and antifungal activities [10,12,13,14]. Few studies have reported the phytochemical composition of *B. thunbergii*: The profile of alkaloids [10,15] and flavonoids contents in roots [3].

The aim of this work is to detail the composition of the phenolic content of leaves of *B. thunbergii*, as well as its antioxidant activity and enzyme inhibitory properties against cholinesterase, amylase, glucosidase, tyrosinase, and lipase. The results here presented may open additional ways to valorize this species as a source of bioactive compounds for the pharmaceutical or food industries.

## 2. Results and Discussion

First of all, MeOH and H_2_O were selected as the extractants, as they are the most common solvents used for the extraction of phenolics from plant materials. A comparison between ultrasound-assisted extraction using an ultrasound bath (Bandelin Sonorex Digital 10P; Sigma-Aldrich, Madrid, Spain) and an ultrasound probe (Qsonica Sonicators, Newtown, CT, USA) was performed. The optimum conditions using the ultrasound bath were 60 min at room temperature. For the ultrasound probe, 5–20 min and 25%–100% power were tested, observing that the highest recovery yields were obtained for 10 min and 50% power; different conditions resulted in lower recovery yields, approximately 20%–30%. These optimum conditions were compared with the recovery obtained with the ultrasound bath, observing similar results. Hence, we selected the ultrasound probe to minimize the time required for sample extraction: 10 min instead of 60 min.

### 2.1. HPLC-MS Analysis of Methanolic and Aqueous Extracts

**Figure 1:**
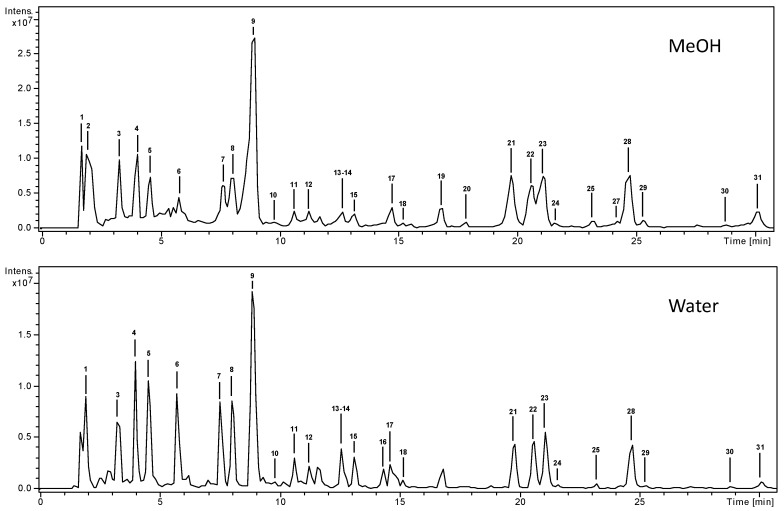
High-performance liquid chromatography-electrospray ionization-mass spectrometry (HPLC-ESI/MS^n^) base peak chromatograms (BPC) of the methanolic and aqueous extracts of *Berberis thunbergii* leaves.

Compounds characterization was carried out by mass spectrometry, using both positive and negative ion modes. The base peak chromatograms of the methanolic and aqueous extracts of *B. thunbergii* leaves are shown in Figure 1 (overlapped chromatograms can be seen in Supplementary Materials). We identified or tentatively characterized 30 compounds; 50% were phenolic acids and approximately 25% flavonoids. All compounds were numbered according to their order of elution (Table 1), keeping the same numeration in both extracts. Among the identified compounds, berberine, rutin, and chlorogenic acid may be cited due to their known bioactivity.

#### 2.1.1. Phenolic Acids

The HPLC profile of the extracts of *B. thunbergii* leaves revealed the highest peak at a retention time of 9 min, which corresponded to chlorogenic acid (compound **9**; identified by comparison with an analytical standard). In other *Berberis* species (*B. microphylla* G. Forst), this compound was the main one [16]. Compounds **7**, **8**, **12**, **13**, and **17** presented deprotonated molecular ions at *m/z* 707 ([2M − H]^−^) or 353 and fragment ions characteristic of caffeoylquinic acids [17]. Several caffeoylquinic acids have been previously reported in *Berberis* species [16,18]. Compound **16,** with [M − H]^−^ ion at *m/z* 705, MS^2^ base peak at *m/z* 513 (neutral loss of 192 Da, which corresponds to quinic acid), and MS^3^ base peak at *m/z* 339 (neutral loss of 174 Da, corresponding to a dehydroquinic acid), was characterized as caffeoylquinic acid dehydrodimer, according to bibliographic data [19]; this compound has not been previously reported in *Berberis* species. With [M − H]^−^ ion at *m/z* 515 and MS^n^ fragment ions at *m/z* 353 and 191, compound **27** was identified as 3,5-dicaffeoylquinic acid [17], previously reported in *B. microphylla* G. Forst [16].

Compounds **2**, **3**, **4**, **5**, and **6** showed [M − H]^−^ ions at *m/z* 371, and fragment ions in MS^2^ and MS^3^ at *m/z* 209 and 191. The fragment ion at *m/z* 209 was caused by the loss of a neutral fragment of a caffeoyl group. The ion at *m/z* 209 refers to the compound glucaric acid (compound **1**). This fragmentation pattern allowed their tentative characterization as caffeoylglucaric acid isomers. A similar phytochemical profile was also found in *B. microphylla* G. Forst [16].

Compounds **19** and **24** displayed deprotonated molecular ions at *m/z* 367 with MS^2^ and MS^3^ base peaks at *m/z* 179 and 135, respectively. These compounds were identified as methyl-caffeoyl-quinate isomers [20].

Compounds **15** and **18** were characterized as coumaroylquinic acid isomers based on its [M − H]^−^ ion at *m/z* 337 and the comparison of its fragmentation pattern with bibliographic data [17]. These compounds have been previously reported in *B. darwinii* [18].

#### 2.1.2. Flavonoids

Previous studies on *B. microphylla* and *B. darwinii* [18] reported the presence of several quercetin glycosides, in agreement with the results observed in *B. thunbergii*. Compound **20** showed [M − H]^−^ ion at *m*/*z* 627, with fragment ions at *m*/*z* 301 and 151 (typical of quercetin), so it was tentatively characterized as a quercetin derivative. Compounds **22** and **23** exhibited [M − H]^−^ ion at *m/z* 463; they suffered the neutral loss of 162 Da (hexoside) to yield quercetin at *m/z* 301, so they were characterized as quercetin-*O*-hexoside. In a similar way, compound **28** was characterized as quercetin-*O*-deoxyhexoside due to the neutral loss of 146 Da to yield quercetin. Compound **25**, with [M − H]^−^ ion at *m/z* 505, suffered the neutral loss of 204 Da (acetylhexoside) to yield quercetin and was tentatively characterized as quercetin-*O*-acetylhexoside [21]. Compound **21** was identified as rutin by comparison with an analytical standard.

Compound **10,** with [M − H]^−^ ion at *m/z* 447, suffered the neutral loss of 162 Da, yielding the aglycone luteolin at *m/z* 285 (characteristic fragment ions at *m/z* 243 and 241), so it was characterized as luteolin-*O*-hexoside.

Compound **14** exhibited an [M − H]^−^ ion at *m/z* 449. It suffered the neutral loss of 162 Da, yielding the aglycone at *m/z* 287, that was characterized as dihydrokaempferol due to its fragment ion at *m/z* 259. Hence, this compound was identified as dihydrokaempferol-*O*-hexoside [22].

Compound **30,** with [M − H]^−^ ion at *m/z* 431, displayed the neutral loss of 146 Da (deoxyhexose), yielding kaempferol aglycone at *m/z* 285 (fragment ion at *m/z* 255), so it was tentatively characterized as kaempferol-*O*-deoxyhexoside [22].

#### 2.1.3. Other Compounds

Compound **26**, with M^+^ ion at *m/z* 551, suffered the neutral loss of 248 Da (malonyl + glucoside), yielding the anthocyanidin named delphinidin at *m/z* 303, and it was characterized as delphinidin malonyl glucose [23]. Delphinidin was also reported in *B. lycium* Royle and B. *microphylla* [24,25], being the most representative anthocyanidin in various species of *Berberis* [26].

Compound **32** was characterized as berberine. This alkaloid showed [M + H]^+^ ion at *m*/*z* 336 and MS^2^ fragmentation ions at *m/z* 321 and *m/z* 293 [27]. Previous studies reported this alkaloid in *B. thunbergii* to be at higher concentrations than in other plants of the same family [15].

Finally, compound **11,** with [M − H]^−^ ion at *m/z* 569, was tentatively characterized as a quinic acid derivative due to the fragments ion characteristic of quinic acid (*m/z* 191 and 173).

### 2.2. Quantification of Phenolic Compounds in All Extracts

Twenty-two compounds were quantified in the analyzed extracts of *B. thunbergii* by HPLC-DAD (Table 2), measuring phenolic acids at 320 nm and flavonoids at 350 nm (see Supplementary Materials).

MeOH and water extracts presented similar phenolic composition, although higher concentration (total amount of phenolics) was found in the methanolic extract. For some individual compounds, higher concentrations were found in the aqueous extract. The results indicated that phenolic acids and flavonoids were the most abundant compounds. Chlorogenic acid presented the highest concentration (101.3 mg g^−1^ DE in MeOH and 90.1 mg g^−1^ DE in water), in agreement with the results reported in another species of *Berberis* (*B. microphylla* G. Forst). However, the concentration of chlorogenic acid in this species was only 0.415 mg g^−1^ and the total concentration of hydroxycinnamic acids was of 1.48 mg g^−1^ [16], lower than the ones found in our studies. In a previous study on 8 species of *Berberis*, the concentration of chlorogenic acid in leaves was determined, obtaining a range of concentrations of 21.8–189.4 mg g^−1^ [28], with only two species presenting higher concentration than the one found in the analyzed extracts of *B. thunbergii*. In the present research, this compound represented almost 50% of the total phenolic acids and 43% of the TIPC (total individual phenolic content, defined as the sum of all individual phenolic concentrations). Thirty per cent of the phenolic acids corresponded to caffeoylglucaric acid isomers. Caffeoylquinic acid isomers and small amounts of other phenolic acids (methyl-caffeoyl-quinate, coumaroylquinic acid isomers) accounted for the remaining 20%. The profile of the phenolic acids obtained in *B. thunbergii* is very similar to that of *B. microphylla* G. Forst, in which caffeoylquinic acids and caffeoylglucaric acids were reported [16].

Leaves were also rich in flavonoids such as quercetin derivatives and kaempferol derivatives (in minor concentrations). Quercetin derivatives represented 77% of the flavonoids quantified in the methanolic extract and around 70% in the aqueous extract. Other species of Berberis (*B. lycium* Royle, *B. microphylla,* and *B. crataegina* DC) also presented quercetin and rutin in their compositions [24,25,29]. TIP in MeOH (236 mg g^−1^ DE) was slightly higher than in the aqueous solution (213 mg g^−1^ DE).

### 2.3. Total Phenolic and Flavonoid Contents

Spectrophotometric methods are considered as an uncertain prediction for the determination of total bioactive components because there is a possibility that other phytochemicals could be participating in the reaction. However, these methods are still common and can provide valuable information for comparison purposes with other works.

The results of total phenolic content (TPC) and total flavonoid content (TFC) assays for *B. thunbergii* extracts are given in Table 3. In accordance with HPLC-MS results, the methanol extract possessed higher concentration of phenolics (216.30 mg GAE/g) and flavonoids (45.85 mg RE/g) than water extract (193.70 and 20.55 mg RE/g), respectively. In previous literature, total bioactive components for several *Berberis* species have been reported. However, most studies were conducted on *Berberis* fruits [30,31,32]. A recent study reported that the methanol extract (80%) of *Berberis orthobotrys* contained more phenolics and flavonoids than the water extract [33], in agreement with our results. Belwal et al. [34] conducted a study on leaves of *Berberis asiatica*, observing that the total phenolic and flavonoid contents were lower than our results. Similarly, *B. vulgaris* (52.54 mg GAE/g) and *B. croatica* (31.16 mg GAE/g) [35] presented much lower phenolic contents than the study extracts of *B. thunbergii.*

### 2.4. Biological Activities

In the present work, antioxidant capacity and enzyme inhibitory effects of *B. thunbergii* extracts were evaluated for their biological effects. The results are summarized in Table 3. The evaluation of antioxidant abilities of plant extracts is of great importance in order to provide novel and safer natural antioxidants for developing functional products. However, no golden standard method has been reported. At this point, several assays could be performed to provide a full picture of the antioxidant effects of plant extracts. In this study, free radical quenching (ABTS and DPPH assays), reducing power (CUPRAC and FRAP), phosphomolybdenum, and metal chelating assays were conducted to examine antioxidant effects of *B. thunbergii* extracts. In all assays, the antioxidant effects in *B. thunbergii* extracts followed the same trend of total bioactive compounds. This was also confirmed by correlation analysis (Table 4). The methanol extract was more active on free radicals than water extract. Additionally, the methanol extract exhibited the best reduction ability in both CUPRAC and FRAP assays. The observed antioxidant abilities of *B. thunbergii* extracts could be explained due to the presence of chlorogenic acid. In accordance with our results, chlorogenic acid has been reported as a good radical scavenger and reductive agent [36,37,38]. In earlier studies, several *Berberis* species have exhibited significant antioxidant effects [30,33,39], thus, members of the genus *Berberis* could be considered as significant sources of natural antioxidants.

Nowadays, novel and safe therapeutic strategies are required to manage several health problems, including type 2 diabetes and obesity. For example, about 425 million people were affected with type 2 diabetes in 2017 [40]. An increasing trend has been reported for obesity [41]. Based on the enzyme inhibition theory, the inhibition of key enzymes is used to alleviate the observed symptoms in diseases [42]. To this end, several compounds are chemically produced as enzyme inhibitors but most of them have undesirable side effects [43]. Hence, there is a growing interest to identify novel, more efficient and less toxic natural enzymatic inhibitors. For these reasons, we tested enzyme inhibitor effects of *B. thunbergii* extracts against cholinesterases (AChE and BChE), tyrosinase, amylase, glucosidase, and lipase. The results are summarized in Table 3. Similarly to the antioxidant results, the methanol extract was more active on all enzymes than water extract. For example, the methanol extract exhibited an inhibitory effect on BChE (0.19 mg GALAE/g), while the water extract was reported as inactive on this enzyme. A similar result was also found for lipase. Additionally, the methanol extract exhibited a significant inhibitory effect on enzymes linked to type 2 diabetes. The observed enzyme inhibitory effect of methanol extract can be attributed to the presence of phenolics, especially chlorogenic acid, and flavonoids such as quercetin and rutin (the main compounds in the analyzed extracts, see Table 2). This approach was also supported by several researchers. For example, Oboh et al. [44] reported that chlorogenic acid exhibited good inhibitory effects on AChE. Furthermore, the anti-amylase and anti-glucosidase effects of chlorogenic acid have been reported [45,46]. Similar observations have been also noted for rutin and quercetin [47,48,49,50]. To the best of our knowledge, the present research is the first scientific report regarding the enzyme inhibitory effect of *B. thunbergii* leaves. From this angle, this study could provide a scientific basis for designing further studies on *B. thunbergii*.

## 3. Materials and Methods

### 3.1. Plant Material

Leaves of *B. thunbergii* were randomly collected from different plants, manually, at the Herbarium of the University of Jaén (Jaén, southeast of Spain; 37°47’18.879”N 3°46’31.583”W, 427 m a.s.l.), in September 2018. Botanical authentication was carried out by the botanist Dr. Carlos Salazar Mendías (Department of Animal Biology, Plant Biology, and Ecology of the University of Jaén, Spain). Only the most intact and fresh leaves were selected. Leaves were washed with ultrapure water and stored in a freezer at −80 °C until use.

### 3.2. Extraction

Extractions were carried out in two different media: Methanol (MeOH; HPLC grade) and water (Milli-Q waters). For the methanol extraction, leaves were lyophilized (ModulyoD/23, Thermo Savant; Waltham, MA, USA) and crushed with a grinder. Extraction was performed as follows: 2.5 g of dry material was extracted with 50 mL MeOH in an ultrasonic liquid processor (Qsonica Sonicators; Newton, CT, USA) with a power of 55 W and a frequency of 20 kHz, for 10 min (using 50% power) at room temperature. Extractions were done in triplicate. After sonication, solutions were filtered through Whatman No.1 filters. The solvent was evaporated under reduced pressure in a Hei-Vap Precision rotary evaporator (Heidolf; Schwabach; Germany) at 40 °C. Dried extracts (DE) were stored at −20 °C until analysis.

On the other hand, the extraction with water was carried out in the following way: 2.5 g of fresh leaves (crushed with a grinder) was extracted with 150 mL H_2_O at 100 °C in a hot plate (C-MAG HS7, IKA; Staufen, Germany) for 30 min. Extractions were done in triplicate. After that, solutions were filtered through Whatman No.1 filters. Finally, the solvent was evaporated under reduced pressure in a rotary evaporator and the dried extracts were stored at −20 °C until analysis.

### 3.3. HPLC Analysis

High-performance liquid chromatography with diode-array and mass spectrometry detection (HPLC-DAD-MS^n^) analysis was performed on an Agilent Series 1100 with a G1315B diode array detector and an ion trap mass spectrometer (Esquire 6000, Bruker Daltonics, Madrid, Spain) with an electrospray interface. A reversed-phase Luna Omega Polar C_18_ analytical column (150 × 3.0 mm; 5 µm particle size; Phenomenex, Madrid, Spain) and a Polar C_18_ Security Guard cartridge (Phenomenex) of 4 × 3.0 mm were used. Detailed conditions were previously reported [51] and given in Supplementary Materials.

5 mg of DE (MeOH) was re-dissolved in 1 mL of MeOH and 5 mg of DE (H_2_O) was re-dissolved in 1 mL of MeOH:H_2_O (10:90; v:v). After filtration through 0.45 µm nylon membrane filters, 10 μL of sample was injected.

Standards of caffeic acid, 3-*O*-caffeoylquinic acid (chlorogenic acid; CAS No. 327-97-9), 4-*O*-caffeoylquinic acid, kaempferol, quercetin, and rutin were obtained from Sigma-Aldrich (Madrid, Spain) and individual stock solutions (500–1000 mg L^−1^) were prepared in MeOH. LC-MS grade acetonitrile (Panreac; Barcelona, Spain) and ultrapure water (Milli-Q Waters purification system; Millipore; Milford, MA, USA) were also used. We prepared calibration curves for caffeic acid, 4-*O*-caffeoylquinic acid, chlorogenic acid, kaempferol, quercetin, and rutin at concentrations 0.5–100 µg mL^−1^ in MeOH. Chromatograms were recorded at 320 nm for caffeic acid, 4-*O*-caffeoylquinic acid and chlorogenic acid, and 350 nm for kaempferol, quercetin, and rutin. Peak area (at the corresponding wavelength) was plotted versus analyte concentration to construct the calibration graphs.

### 3.4. Assays for Total Phenolic and Flavonoid Contents

With reference to our earlier report [52], total bioactive components, namely total phenolic (TPC) and flavonoid (TFC) contents, were measured by spectrophotometric assays. The obtained results were reported as standard equivalents of gallic acid for phenolics and rutin for flavonoids. Details for the protocols are provided in Supplementary Materials.

### 3.5. Determination of Antioxidant and Enzyme Inhibitory Effects

The in vitro enzyme inhibitory effects of *B. thunbergii* extracts on five enzymes (lipase, α-amylase, α-glucosidase, cholinesterases, and tyrosinase) were evaluated as previously reported [52,53]. The enzyme inhibitory actions were assessed as kojic acid equivalents (KAE) for tyrosinase, galantamine equivalents (GALAE) for acetyl cholinesterase (AChE) and butyryl cholinesterase (BChE), acarbose equivalents (ACAE) for α-amylase and α-glucosidase, and orlistat equivalents (OE) for lipase. Details for the protocols are provided in Supplementary Materials.

Regarding the antioxidant capacity of *B. thunbergii* extracts, different experiments such as ferrous ion chelating, phosphomolybdenum, and radicals scavenging tests (FRAP, ABTS, CUPRAC, and DPPH) were spectrophotometrically screened. The findings are given as standard compounds equivalents of Trolox and EDTA. The assay methods were described in our earlier work [52]. Details for the protocols are provided in Supplementary Materials.

### 3.6. Statistical Analysis

The analysis were performed in triplicate. The results were given as mean and standard deviation. The differences in the extracts were investigated by using student *t*-test (*p* < 0.05) and this test was performed in Xlstat 2018. The relationship between biological activities and total bioactive compounds based on the estimation of Pearson’s correlation coefficients were conducted. The correlation analysis was performed by using R software v. 3.6.1.

## 4. Conclusions

We have analyzed the phenolic profile of *B. thunbergii* DC. leaves and quantified the most abundant compounds. Two extracts (MeOH and water) were prepared, observing a higher recovery yield in the MeOH extract. The most abundant compounds in both extracts were chlorogenic acid (101.3 mg g^−1^ DE in MeOH extract), followed by other caffeoylquinic acids and caffeoylglucaric acids; quercetin glycosides were also quantified. Although both extracts exhibited potent antioxidant activity, the MeOH extract was the most potent, probably due to the highest TIPC, although all compounds may interact in a synergetic way. The enzyme inhibitory effect of the extracts was tested, observing that the MeOH extract was active against acetylcholinesterase, butyrylcholinesterase, tyrosinase, amylase, glucosidase, and lipase. However, the aqueous extract was inactive against butyrylcholinesterase and lipase, showing also less inhibition than the MeOH extract towards the other enzymes. In general, MeOH extract presented a high concentration of phenolic compounds and potent bioactivity, making it a suitable candidate for further analysis (isolation and bioactivity of the main compounds) with potential applications in the pharmaceutical and food industry (preparation of novel supplements or nutraceuticals).

## Figures and Tables

**Table 1:** Characterization of phytochemicals found in extracts of *Berberis thunbergii* by HPLC-/ESI-MS^n^.

No.	t*_R _*(min)	[M − H]^− ^*m/z*	*m*/*z* (% Base Peak)	Assigned Identification	Berberis MeOH	Berberis Water
**1**	1.7	209	MS2 [209]: 191 (100), 173 (2)	Glucaric acid		✓
**2**	2.0	371	MS2 [371]: 353 (11), 209 (100), 191 (29) MS3 [371→209]: 191 (100), 173 (6)	Caffeoylglucaric acid isomer	✓	
**3**	3.3	371	MS2 [371]: 353 (8), 209 (100), 191 (35) MS3 [371→209]: 191 (100), 173 (3), 147 (7), 129 (2)	Caffeoylglucaric acid isomer	✓	✓
**4**	4.1	371	MS2 [371]: 353 (15), 209 (100), 191 (43) MS3 [371→209]: 191 (100), 173 (5), 147 (6), 129 (2)	Caffeoylglucaric acid isomer	✓	✓
**5**	4.6	371	MS2 [371]: 353 (8), 209 (100), 191 (21) MS3 [371→209]: 191 (100), 173 (13)	Caffeoylglucaric acid isomer	✓	✓
**6**	5.7	371	MS2 [371]: 353 (13), 209 (100), 191 (33) MS3 [371→209]: 191 (100), 173 (4)	Caffeoylglucaric acid isomer	✓	✓
**7**	7.7	707	MS2 [707]: 353 (100), 309 (5) MS3 [707→353]: 191 (100), 179 (16)	Caffeoylquinic acid	✓	✓
**8**	8.0	707	MS2 [707]: 353 (100) MS3 [707→353]: 335 (3), 191 (100)	Caffeoylquinic acid	✓	✓
**9**	9.0	707	MS2 [707]: 353 (100) MS3 [707→353]: 191 (100), 179 (3)	Chlorogenic acid*	✓	✓
**10**	9.8	447	MS2 [447]: 285 (100) MS3 [447→285]: 257 (100), 243 (70), 241 (88), 175 (61)	Luteolin-*O*-hexoside	✓	✓
**11**	10.6	569	MS2 [569]: 389 (8), 371 (100), 327 (8), 265 (12), 173 (8) MS3 [569→371]: 191 (36), 173 (97), 129 (100)	Quinic acid derivative	✓	✓
**12**	11.2	707	MS2 [707]: 353 (100) MS3 [707→353]: 191 (100), 179 (6), 173 (4)	Caffeoylquinic acid	✓	✓
**13**	12.5	353	MS2 [353]: 191 (100), 179 (14), 173 (4), 129 (3) MS3 [353→191]: 173 (79), 147 (30), 129 (60), 103 (100)	Caffeoylquinic acid	✓	✓
**14**	12.6	449	MS2 [449]: 287 (100), 269 (31), 259 (38) MS3 [449→287]: 259 (100), 243 (9), 125 (10)	Dihydrokaempferol-*O*-hexoside	✓	✓
**15**	13.1	337	MS2 [337]: 191 (100), 173 (3), 163 (5) MS3 [337→191]: 127 (100)	Coumaroylquinic acid isomer	✓	✓
**16**	14.3	705	MS2 [705]: 513 (100), 487 (4), 339 (7) MS3 [705→513]: 451 (3), 339 (100), 295 (4) MS4 [705→513→339]: 295 (100), 223 (18)	Caffeoylquinic acid dehydrodimer		✓
**17**	14.7	353	MS2 [353]: 335 (100), 201 (30), 179 (26), 173 (70) MS3 [353→335]: 229 (51), 201 (34), 173 (100), 129 (80) MS4 [353→335→173]: 129 (100)	Caffeoylquinic acid	✓	✓
**18**	15.2	337	MS2 [337]: 191 (100), 173 (3), 163 (5) MS3 [337→191]: 127 (100)	Coumaroylquinic acid isomer	✓	✓
**19**	16.7	367	MS2 [367]: 191 (31), 179 (100), 135 (53) MS3 [367→179]: 135 (100)	Methyl-caffeoyl-quinate	✓	
**20**	17.8	627	MS2 [627]: 473 (11), 447 (100), 301 (7) MS3 [627→447]: 301 (100), 151 (7) MS4 [627→447→301]: 271 (63), 179 (80), 151 (100)	Quercetin derivative	✓	
**21**	19.7	609	MS2 [609]: 302 (15), 301 (100), 300 (10) MS3 [609→301]: 255 (30), 179 (100), 151 (60) MS4 [609→301→179]: 153 (31), 151 (100)	Rutin *	✓	✓
**22**	20.5	463	MS2 [463]: 301 (100) MS3 [463→301]: 255 (28), 179 (100), 151 (64) MS4 [463→301→179]: 151 (100), 107 (73)	Quercetin-*O*-hexoside	✓	✓
**23**	21.0	463	MS2 [463]: 301 (100) MS3 [463→301]: 255 (34), 179 (100), 151 (68) MS4 [463→301→179]: 151 (100), 107 (62)	Quercetin-*O*-hexoside	✓	✓
**24**	21.5	367	MS2 [367]: 335 (89), 179 (100), 161 (77), 135 (21) MS3 [367→179]: 135 (100)	Methyl-caffeoyl-quinate	✓	✓
**25**	23.1	505	MS2 [505]: 463 (13), 343 (3), 301 (100) MS3 [505→301]: 271 (36), 255 (42), 179 (80), 151 (100)	Quercetin-*O*-acetylhexoside	✓	✓
**26**	23.1	551 (+)	MS2 [551]: 533 (3), 303 (100) MS3 [551→303]: 257 (100), 153 (55)	Delphinidin malonyl glucoside	✓	✓
**27**	24.0	515	MS2 [515]: 353 (100), 191 (6) MS3 [515→353]: 191 (100), 179 (38), 135 (11)	3,5-Dicaffeoylquinic acid	✓	
**28**	24.6	447	MS2 [447]: 301 (100), 179 (3), 151 (3) MS3 [447→301]: 271 (23), 255 (17), 179 (54), 151 (100) MS4 [447→301→151]: 107 (100)	Quercetin-*O*-deoxyhexoside	✓	✓
**29**	25.2	481	MS2 [481]: 345 (6), 327 (100), 217 (11), 153 (21) MS3 [481→327]: 217 (100), 189 (8), 165 (6) MS4 [481→327→217]: 189 (100)	Unknown	✓	✓
**30**	28.7	431	MS2 [431]: 285 (100), 255 (7) MS3 [431→285]: 267 (85), 257 (55), 255 (100), 199 (49), 163 (65)	Kaempferol-*O*-deoxyhexoside	✓	✓
**31**	30.0	613	MS2 [613]: 503 (100), 451 (13), 393 (28), 379 (5), 341 (15) MS3 [613→503]: 341 (90), 393 (100), 379 (13) MS4 [613→503→393]: 284 (31), 269 (100), 229 (40)	Unknown	✓	✓
**32**	33.8	336 (+)	MS2 [336]: 321 (100), 293 (11), 292 (26) MS3 [336→321]: 292 (100)	Berberine	✓	✓

* Identified with analytical standards.

**Table 2:** Quantification of compounds in extracts of *Berberis thunbergii *in methanol (MeOH) and water. Values (mg g^−1^ dried extract; DE) are mean ± SD of three parallel measurements.

*N°.*		MeOH	Water
***Phenolic Acids***			
**2**	Caffeoylglucaric acid isomer	38 ± 4	-
**3**	Caffeoylglucaric acid isomer	5.9 ± 0.1 ^b^	12.2 ± 0.3 ^a^
**4**	Caffeoylglucaric acid isomer	10.97 ± 0.7 ^b^	15 ± 1 ^a^
**5**	Caffeoylglucaric acid isomer	5.7 ± 0.3 ^b^	14.6 ± 0.7 ^a^
**6**	Caffeoylglucaric acid isomer	8.89 ± 0.01 ^b^	16.19 ± 0.03 ^a^
**7**	Caffeoylquinic acid	8.3 ± 0.3 ^b^	14.9 ± 0.5 ^a^
**8**	Caffeoylquinic acid	24 ± 1 ^b^	28 ± 2 ^a^
**9**	Chlorogenic acid	101.3 ± 0.4 ^a^	90.1 ± 0.3 ^b^
**12**	Caffeoylquinic acid	1.216 ± 0.009 ^b^	1.76 ± 0.01 ^a^
**15**	Coumaroylquinic acid isomer	0.94 ± 0.01 ^b^	1.43 ± 0.02 ^a^
**16**	Caffeoylquinic acid dehydrodimer	-	0.73 ± 0.03
**17**	Caffeoylquinic acid	4.4 ± 0.3 ^a^	3.6 ± 0.2 ^b^
**18**	Coumaroylquinic acid isomer	0.17 ± 0.02 ^b^	0.28 ± 0.05 ^a^
**19**	Methyl-caffeoyl-quinate	0.43 ± 0.03	-
**24**	Methyl-caffeoyl-quinate	0.21 ± 0.01 ^a^	0.16 ± 0.01 ^b^
**Total**		**210 ± 4 ^a^**	**199 ± 2 ^b^**
***Flavonoids***			
**20**	Quercetin derivative	0.36 ± 0.02	-
**21**	Rutin	6.0 ± 0.2 ^a^	4.2 ± 0.2 ^b^
**22**	Quercetin-*O*-hexoside	5.23 ± 0.06 ^a^	2.62 ± 0.04 ^b^
**23**	Quercetin-*O*-hexoside	7.92 ± 0.03 ^a^	4.04 ± 0.2 ^b^
**25**	Quercetin-*O*-acetylhexoside	0.57 ± 0.02 ^a^	0.30 ± 0.02 ^b^
**28**	Quercetin-*O*-deoxyhexoside	6.2 ± 0.2 ^a^	2.9 ± 0.1 ^b^
**30**	Kaempferol-*O*-deoxyhexoside	0.081 ± 0.001 ^a^	0.043 ± 0.001 ^b^
**Total**		**26.4 ± 0.3 ^a^**	**14.1 ± 0.3 ^b^**
**TIPC**		**236 ± 4 ^a^**	**213 ± 2 ^b^**

Different superscripts (^a^ and ^b^) indicate significant differences in the extracts (*p *< 0.05).

**Table 3:** Total bioactive components, antioxidant, and enzyme inhibitory properties of the tested extracts.

Assays	MeOH	Water
***Total bioactive components***		
TPC (mg GAE/g)	216 ± 6 ^a^	194 ± 1 ^b^
TFC (mg RE/g)	46 ± 1 ^a^	20.6 ± 0.5 ^b^
***Antioxidant assays***		
DPPH (mg TE/g)	429 ± 6 ^a^	360 ± 20 ^b^
ABTS (mg TE/g)	450 ± 7 ^a^	352 ± 4 ^b^
CUPRAC (mg TE/g)	1232 ± 5 ^a^	1120 ± 20 ^b^
FRAP (mg TE/g)	620 ± 10 ^a^	549 ± 6 ^b^
Phosphomolybdenum (mmol TE/g)	5.7 ± 0.3 ^a^	5.40 ± 0.05 ^a^
Metal Chelating (mg EDTAE/g)	4.54 ± 0.01 ^a^	2.35 ± 0.01 ^b^
***Enzyme inhibition assays***		
AChE inhibition (mg GALAE/g)	1.9 ± 0.2 ^a^	1.15 ± 0.01 ^b^
BChE inhibition (mg GALAE/g)	0.19 ± 0.05	ni
Tyrosinase inhibition (mg KAE/g)	33 ± 3 ^a^	29.8 ± 0.8 ^b^
Amylase inhibition (mmol ACAE/g)	0.68 ± 0.01 ^a^	0.10 ± 0.01 ^b^
Glucosidase (mmol ACAE/g)	2.54 ± 0.01 ^a^	2.22 ± 0.01 ^b^
Lipase (mg OE/g)	54 ± 5	ni

Values (expressed in mg or mmol per g DE) are means ± SD of three parallel measurements. TPC: Total phenolic content; TFC: Total flavonoid content; GAE: Gallic acid equivalent; RE: Rutin equivalent; TE: Trolox equivalent; EDTAE: EDTA equivalent; GALAE: Galatamine equivalent; KAE: Kojic acid equivalent; ACAE: Acarbose equivalent; OE: Orlistat equivalent; ni: No inhibition. Different superscripts (^a^ and ^b^) indicate significant differences in the extracts (*p* < 0.05).

**Table 4:** Correlation coefficients between total bioactive compounds and biological activities (Pearson correlation coefficient (*R*), *p* < 0.05).

	DPPH	ABTS	CUPRAC	FRAP	PHO^a^	MCA^b^	AChE	BChE	Tyrosinase	Amylase	Glucosidase	Lipase
TPC	0.91	0.97	0.95	0.95	0.48	0.96	0.99	0.92	0.69	0.96	0.96	0.95
TFC	0.96	0.99	0.98	0.98	0.66	0.99	0.95	0.98	0.81	0.99	0.99	0.99

^a^ Phosphomolybdenum assay. ^b^ Metal chelating assay.

## Supplementary Materials

The following are available online. Detailed protocols for chromatographic analysis, total phenolic and flavonoid contents, antioxidant and enzyme inhibitory assays; HPLC-MS and HPLC-UV chromatograms of *Berberis* extracts.
