# Supplementary material for: Phenolic Characterization, Antioxidant Activity, and Enzyme Inhibitory Properties of Berberis thunbergii DC. Leaves: A Valuable Source of Phenolic Acids
Source: Molecules. 2019 Nov 17;24(22):4171. doi: 10.3390/molecules24224171 (PMC6891573; doi:10.3390/molecules24224171)

Phenolic Characterization, Antioxidant Activity, and Enzyme Inhibitory Properties of *Berberis thunbergii* DC. Leaves: a Valuable Source of Phenolic Acids

María del Pilar Fernández-Poyatos[a], Antonio Ruiz-Medina[a], Gokhan Zengin[b] and Eulogio J. Llorent-Martínez[a,*]

Supplementary Materials

3.3. HPLC Analysis Conditions

For HPLC analysis, 5 mg of dried extract (DE) was re-dissolved in 1 ml of methanol, filtered through 0.45 µm PTFE membrane filters, and 10 µL of the solution was injected. The HPLC system was an Agilent Series 1100, composed of a vacuum degasser, an autosampler, a binary pump, and a G1315B diode array detector (Agilent Technologies, Santa Clara, CA, USA). We used a reversed phase Luna Omega Polar C_{18} analytical column of 150 x 3.0 mm and 5 µm particle size (Phenomenex, Torrance, CA, USA) and a Polar C_{18} Security Guard cartridge (Phenomenex) of 4 x 3.0 mm. The best separation was achieved with a mobile phase of water-formic acid (100:0.1, v/v) and CH_3CN. The following program was used: a) initial mobile phase, 10% CH_3CN; b) linear increase from 10% to 25 % CH_3CN (0-25 min); c) 25 % CH_3CN (25-30 min); d) linear increase from 25 % to 50 % CH_3CN (30-40 min); e) linear increase from 50 % to 100 % CH_3CN (40-42 min); f) 100 % CH_3CN (42-47 min). Then, CH_3CN percentage was returned to the initial mobile phase, with a 7 min stabilization time. The flow rate was 0.4 ml min^{-1}.

The HPLC system was connected to an ion trap mass spectrometer (Esquire 6000, Bruker Daltonics, Billerica, MA, USA) equipped with an electrospray interface. The scan range was at m/z 100–1200 with a speed of 13,000 Da/s. The ESI conditions were: drying gas (N_2) flow rate and temperature, 10 mL/min and 365 °C; nebulizer gas (N_2) pressure, 50 psi; capillary

voltage, 4500 V; capillary exit voltage, -117.3 V. We used the auto MS^n mode for the acquisition of MS^n data, with isolation width of 4.0 m/z, and fragmentation amplitude of 0.6 V (MS^n up to MS^4).

3.4. Assays for Total Phenolic and Flavonoid Contents

The total phenolic content was determined by employing the methods given in the literature with some modification. Sample solution (0.25 mL) was mixed with diluted Folin–Ciocalteu reagent (1 mL, 1:9, v/v) and shaken vigorously. After 3 min, Na_2CO_3 solution (0.75 mL, 1%) was added and the sample absorbance was read at 760 nm after a 2 h incubation at room temperature. The total phenolic content was expressed as milligrams of gallic acid equivalents (mg GAE/g extract) (Uysal et al., 2017).

The total flavonoid content was determined using the $AlCl_3$ method. Briefly, sample solution (1 mL) was mixed with the same volume of aluminum trichloride (2%) in methanol. Similarly, a blank was prepared by adding sample solution (1 mL) to methanol (1 mL) without $AlCl_3$. The sample and blank absorbances were read at 415 nm after a 10 min incubation at room temperature. The absorbance of the blank was subtracted from that of the sample. Rutin was used as a reference standard and the total flavonoid content was expressed as milligrams of rutin equivalents (mg RE/g extract) (Uysal et al., 2017).

3.5. Determination of Antioxidant and Enzyme Inhibitory Effects

Antioxidant (DPPH and ABTS radical scavenging), reducing power (CUPRAC and FRAP), phosphomolybdenum and metal chelating (ferrozine method) and enzyme inhibitory activities (cholinesterase (Elmann’s method), tyrosinase (dopachrome method), α-amylase (iodine/potassium iodide method), α -glucosidase (chromogenic PNPG method) and pancreatic

lipase (*p*-nitrophenyl butyrate (p-NPB) method) were determined using the methods previously described by Uysal et al. (2017) and Grochowski et al. (2017).

For the DPPH (1,1-diphenyl-2-picrylhydrazyl) radical scavenging assay: Sample solution was added to 4 mL of a 0.004% methanol solution of DPPH. The sample absorbance was read at 517 nm after a 30 min incubation at room temperature in the dark. DPPH radical scavenging activity was expressed as milligrams of trolox equivalents (mg TE/g extract).

For ABTS (2,2′-azino-bis(3-ethylbenzothiazoline) 6-sulfonic acid) radical scavenging assay: Briefly, $ABTS^+$ was produced directly by reacting 7 mM ABTS solution with 2.45 mM potassium persulfate and allowing the mixture to stand for 12–16 in the dark at room temperature. Prior to beginning the assay, ABTS solution was diluted with methanol to an absorbance of 0.700 ± 0.02 at 734 nm. Sample solution was added to ABTS solution (2 mL) and mixed. The sample absorbance was read at 734 nm after a 30 min incubation at room temperature. The ABTS radical scavenging activity was expressed as milligrams of trolox equivalents (mg TE/g extract).

For CUPRAC (cupric ion reducing activity) activity assay: Sample solution was added to premixed reaction mixture containing $CuCl_2$ (1 mL, 10 mM), neocuproine (1 mL, 7.5 mM) and NH_4Ac buffer (1 mL, 1 M, pH 7.0). Similarly, a blank was prepared by adding sample solution (0.5 mL) to premixed reaction mixture (3 mL) without $CuCl_2$. Then, the sample and blank absorbances were read at 450 nm after a 30 min incubation at room temperature. The absorbance of the blank was subtracted from that of the sample. CUPRAC activity was expressed as milligrams of trolox equivalents (mg TE/g extract).

For FRAP (ferric reducing antioxidant power) activity assay: Sample solution was added to premixed FRAP reagent (2 mL) containing acetate buffer (0.3 M, pH 3.6), 2,4,6-tris(2-pyridyl)-S-triazine (TPTZ) (10 mM) in 40 mM HCl and ferric chloride (20 mM) in a ratio of

10:1:1 (v/v/v). Then, the sample absorbance was read at 593 nm after a 30 min incubation at room temperature. FRAP activity was expressed as milligrams of trolox equivalents (mg TE/g extract).

For phosphomolybdenum method: Sample solution was combined with 3 mL of reagent solution (0.6 M sulfuric acid, 28 mM sodium phosphate and 4 mM ammonium molybdate). The sample absorbance was read at 695 nm after a 90 min incubation at 95 ℃. The total antioxidant capacity was expressed as millimoles of trolox equivalents (mmol TE/g extract).

For metal chelating activity assay: Briefly, sample solution was added to $FeCl_2$ solution (0.05 mL, 2 mM). The reaction was initiated by the addition of 5 mM ferrozine (0.2 mL). Similarly, a blank was prepared by adding sample solution (2 mL) to $FeCl_2$ solution (0.05 mL, 2 mM) and water (0.2 mL) without ferrozine. Then, the sample and blank absorbances were read at 562 nm after 10 min incubation at room temperature. The absorbance of the blank was sub-tracted from that of the sample. The metal chelating activity was expressed as milligrams of EDTA (disodium edetate) equivalents (mg EDTAE/g extract).

For Cholinesterase (ChE) inhibitory activity assay: Sample solution (was mixed with DTNB (5,5-dithio-bis(2-nitrobenzoic) acid, Sigma, St. Louis, MO, USA) (125 µL) and AChE (acetylcholines-terase (Electric ell acetylcholinesterase, Type-VI-S, EC 3.1.1.7,Sigma)), or BChE (butyrylcholinesterase (horse serum butyrylcholinesterase, EC 3.1.1.8, Sigma)) solution (25 µL) in Tris–HCl buffer (pH 8.0) in a 96-well microplate and incubated for 15 min at 25 ℃. The reaction was then initiated with the addition of acetylthiocholine iodide (ATCI, Sigma) or butyrylthiocholine chloride (BTCl, Sigma) (25 µL). Similarly, a blank was prepared by adding sample solution to all reaction reagents without enzyme (AChE or BChE) solution. The sample and blank absorbances were read at 405 nm after 10 min incubation at 25 ℃. The absorbance

of the blank was subtracted from that of the sample and the cholinesterase inhibitory activity was expressed as galanthamine equivalents (mg GALAE/g extract).

For Tyrosinase inhibitory activity assay: Sample solution was mixed with tyrosinase solution (40 μL, Sigma) and phosphate buffer (100 μL, pH 6.8) in a 96-well microplate and incubated for 15 min at 25 ℃. The reaction was then initiated with the addition of L-DOPA (40 μL, Sigma). Similarly, a blank was prepared by adding sample solution to all reaction reagents without enzyme (tyrosinase) solution. The sample and blank absorbances were read at 492 nm after a 10 min incubation at 25 ℃. The absorbance of the blank was subtracted from that of the sample and the tyrosinase inhibitory activity was expressed as kojic acid equivalents (mg KAE/g extract).

For α-amylase inhibitory activity assay: Sample solution was mixed with α-amylase solution (ex-porcine pancreas, EC 3.2.1.1, Sigma) (50 μL) in phosphate buffer (pH 6.9 with 6 mM sodium chloride) in a 96-well microplate and incubated for 10 min at 37 ℃. After pre-incubation, the reaction was initiated with the addition of starch solution (50 μL, 0.05%). Similarly, a blank was prepared by adding sample solution to all reaction reagents without enzyme (α-amylase) solution. The reaction mixture was incubated 10 min at 37 ℃. The reaction was then stopped with the addition of HCl (25 μL, 1 M). This was followed by addition of the iodine-potassium iodide solution (100 μL). The sample and blank absorbances were read at 630 nm. The absorbance of the blank was subtracted from that of the sample and the α-amylase inhibitory activity was expressed as acarbose equivalents (mmol ACAE/g extract).

For α-glucosidase inhibitory activity assay: Sample solution was mixed with glutathione (50 μL), α-glucosidase solution (from Saccharomyces cerevisiae, EC 3.2.1.20, Sigma) (50 μL) in phosphate buffer (pH 6.8) and PNPG (4-N-trophenyl-α-D-glucopyranoside, Sigma) (50 μL) in a 96-well microplate and incubated for 15 min at 37 ℃. Similarly, a blank was prepared by

adding sample solution to all reaction reagents without enzyme (α-glucosidase) solution. The reaction was then stopped with the addition of sodium carbonate (50 µL, 0.2 M). The sample and blank absorbances were read at 400 nm. The absorbance of the blank was subtracted from that of the sample and the α-glucosidase inhibitory activity was expressed as acarbose equivalents (mmol ACAE/g extract).

Porcine pancreatic lipase (type-II) activity was performed using *p*-nitrophenyl butyrate (p-NPB) as substrate (Roh and Jung, 2012). Enzyme solution (1 mg/mL) was prepared in 50 mM Tris-HCl (pH 8.0). Fraction solution (25 µL) was mixed with a lipase solution (50 µL) in a 96-well microplate and incubated for 20 minutes at 25 ℃. The reaction was initiated with the addition of p-NPB (5 mM, 50 µL). Similarly, a blank sample (prepared in the same manner but without the extract) was prepared for each of the samples and analysed according to this procedure. Results were expressed as orlistat equivalents (mg OE/g extract).

References

Uysal, S.; Zengin, G.; Locatelli, M.; Bahadori, M.B.; Mocan, A.; Bellagamba, G.; De Luca, E.; Mollica, A.; Aktumsek, A. Cytotoxic and enzyme inhibitory potential of two *Potentilla* species (*P. speciosa* L. and *P. reptans* Willd.) and their chemical composition. *Front. Pharmacol.* **2017**, *8*, 290.

Grochowski, D.M.; Uysal, S.; Aktumsek, A.; Granica, S.; Zengin, G.; Ceylan, R.; Locatelli, M.; Tomczyk, M. *In vitro* enzyme inhibitory properties, antioxidant activities, and phytochemical profile of *Potentilla thuringiaca*. *Phytochem. Lett.* **2017**, *20*, 365–372.

Chromatograms.

Figure S1. HPLC-ESI/MS^n base peak chromatograms (BPC) of the methanolic and aqueous extracts of *Berberis thunbergii* leaves.

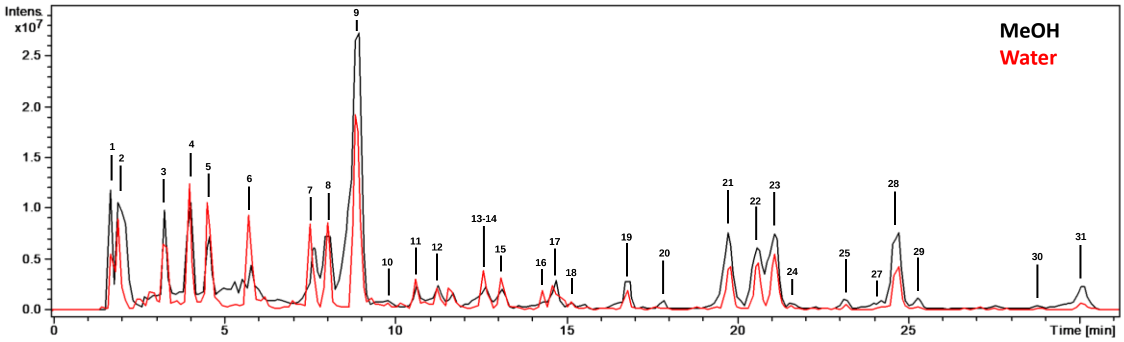

Figure S2. UV chromatograms of the methanolic and aqueous extracts of *Berberis thunbergii* leaves at 320 and 350 nm.

UV- chromatograms at 320 nm; used for the quantification of phenolic acids. Time windows have been selected for a better comparison of the chromatograms.

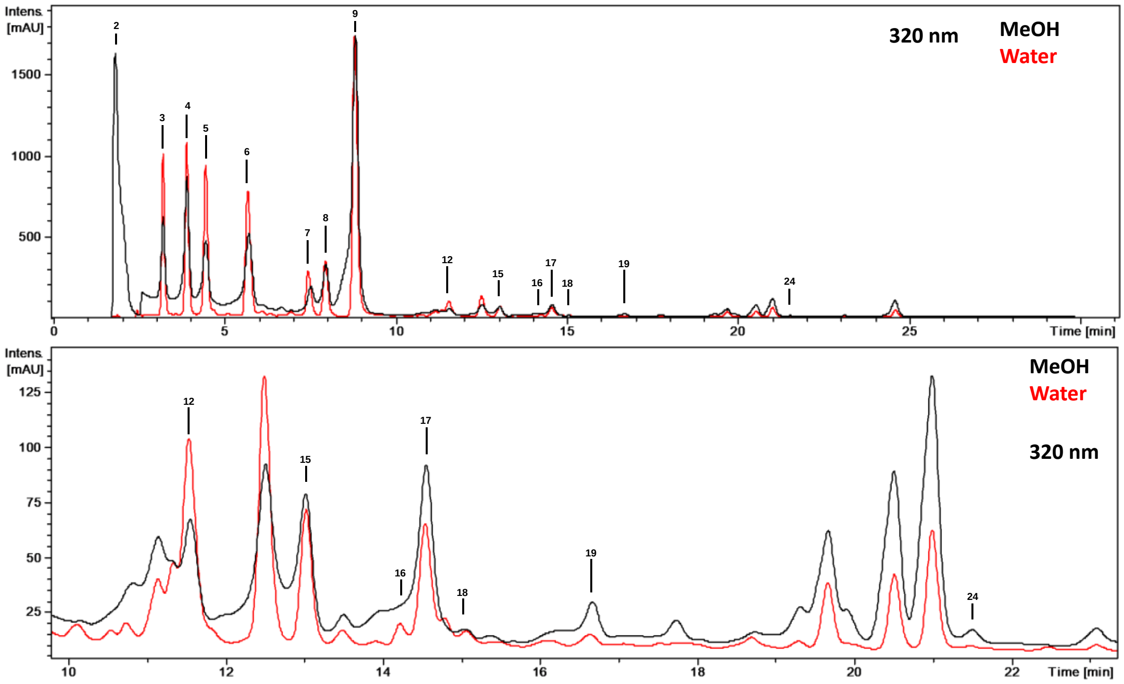

UV- chromatogram at 350 nm; used for the quantification of flavonoids. Time window has been zoomed to show only the part of the chromatogram where these compounds appear.

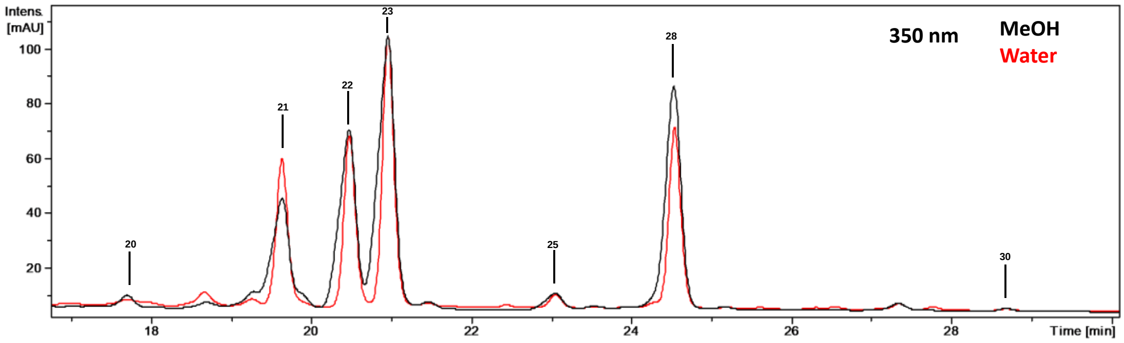

Supplement: Supplementary file 1 [file molecules-24-04171-s001.pdf]
